# Microcystic serous cystadenoma of the pancreas causing biliary obstruction: a case report and review of the literature

**DOI:** 10.1093/jscr/rjae105

**Published:** 2024-03-08

**Authors:** Sydney Bland, William Thompson III

**Affiliations:** Department of General Surgery, Brookwood Baptist Health, Birmingham AL 35211, United States; Alabama Oncology, Grandview Medical Center, Birmingham AL 35243, United States

**Keywords:** microcystic serous cystadenoma, biliary obstruction, Whipple surgery

## Abstract

Cystic tumors account for 15% of pancreatic tumors. Of these, serous microcystic adenomas represent 1–2% of pancreatic exocrine neoplasms. While typically benign, a small percentage possess malignant potential. Given imaging improvements, serous cystadenomas are being identified more frequently. A 63-year-old female was admitted with complaints of jaundice and unintentional weight loss. Abdominal computed tomography scan showed a 16 cm obstructive pancreatic mass near the porta hepatis region. Endoscopic ultrasonography and fine needle aspiration biopsy indicated a large pancreatic head cystic mass favoring serous microcystadenoma causing biliary and some pyloric obstruction. Malignant potential could not be ruled out because of size and symptoms. A pylorus-preserving pancreaticoduodenectomy revealed a cystic tumor invading the pancreatic duct and adhering to the duodenum of the pancreatic head. Pathology confirmed a 15 cm benign pancreatic serous cystadenoma. Although most serous cystadenomas are benign, surgical resection was prudent given the size, symptoms, and adjacent organ involvement.

## Introduction

Cystic neoplasms of the pancreas can be divided into four groups: serous cystadenoma, mucinous cystadenoma/cystadenocarcinoma, intraductal papillary mucinous tumor (IPMN), and rare cystic neoplasms [1]. The World Health Organization (WHO) has classified serous cystadenomas into microcystic and oligocystic variants [1]. Serous microcystic adenomas commonly present during the seventh to eighth decades with a predilection in females and range from 1–26 cm in size [2]. Since the majority of these are often small at time of diagnosis, many do not exhibit symptoms. Less frequently, patients may experience symptoms as the tumor grows including abdominal pain, nausea, vomiting, or weight loss [3]. One-third of tumors are often incidentally identified to involve the pancreatic body and tail [3]. The majority of these are benign and only a small handful of serous cystadenocarcinomas have been reported [4]. Patients who are asymptomatic with small tumors are historically managed conservatively. However, resection has often been the standard of care given a large size, presence of symptoms, or if unable to adequately diagnose and/or differentiate serous versus mucinous cystic neoplasm; the latter with a higher potential for malignancy [4]. In this study, we present a giant serous microcystic cystadenoma with biliary obstruction and review the literature regarding diagnosis and treatment.

## Case presentation

A 63-year-old African American female, with a history of hypertension and developmental delay, presented with complaints of jaundice and unintentional weight loss. The patient did not have any significant family history and no prior admissions. General examination was normal. Patient with a total bilirubin of 20.2 mg/dL (normal range, 0.3–1.2 mg/dL) and findings of an obstructive pancreatic mass on computed tomography (CT) abdomen pelvis. Endoscopic Ultrasound (EUS) was performed with identification of a large classic serous microcystadenoma of the pancreatic head causing biliary obstruction and some degree of pyloric obstruction without identification of any malignant features. Using a 22 gauge needle, an endoscopic ultrasound-guided fine-needle-aspiration biopsy (EUS-FNAB) provided two samplings of tissue. Next, an endoscopic retrograde cholangiopancreatography (ERCP) was performed with difficulty and cannulation of only the pancreatic duct. The gastroenterologist was unable to cannulate the common bile duct during ERCP due to altered anatomy secondary to the massive cyst compressing the upper gastrointestinal tract. A fair amount of dark sludge/bile was identified in the duodenum. Pathology results from EUS-FNAB of the pancreatic head cystic mass were consistent with mostly fibrous pancreatic tissue with focal areas containing serous-type epithelium with mild atypia, favoring serous cystadenoma and focal benign gastric epithelium negative for dysplasia, or malignancy. Patient subsequently underwent percutaneous transhepatic catheter (PTC) placement.

**Figure 1:**
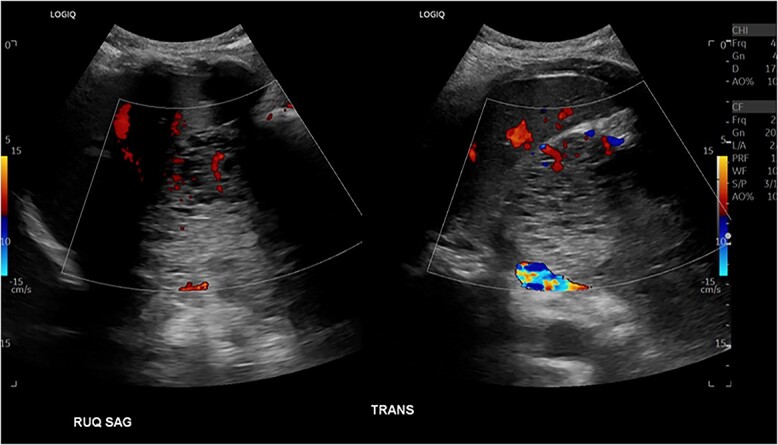
Abdominal US showing a nonspecific mass in RUQ with numerous microcysts.

Patient presented one month later to the hospital from preadmission testing due to abnormal renal function. The patient endorsed dark urine, persistent unintentional weight loss, and decreased appetite with subsequent improvement of jaundice since PTC placement. She presented with abnormal serum biochemistry results: creatinine, 11.90 mg/dL [previously, 1.8 mg/dL] (normal range, 0.7–1.3 mg/dL); potassium, 2.9 mEq/L (normal range, 3.5–5.0 mEq/L); alkaline phosphatase, 142 U/L (normal range, 36–92 U/L); total bilirubin, 2.7 mg/dL (normal range, 0.3–1.2 mg/dL); direct bilirubin, 2.1 mg/dL (normal range, 0–0.3 mg/dL); aspartate aminotransferase (AST), 68 U/L (normal range, 0–35 U/L); alanine aminotransferase (ALT), 135 U/L (normal range, 0–35 U/L). Patient’s cancer antigen 19–9 was 2.2 U/mL (normal range, 0–37 U/mL). Abdominal ultrasonography showed a 13.0 × 9.4 × 8.3 cm mass in the right upper quadrant with numerous microcysts (Fig. 1).

**Figure 2:**
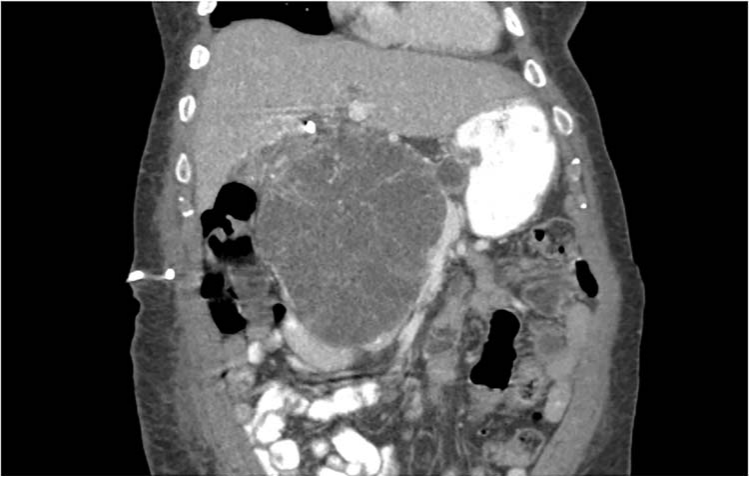
Coronal CT showing a large heterogeneous mass in the pancreatic head with typical honeycomb feature.

**Figure 3:**
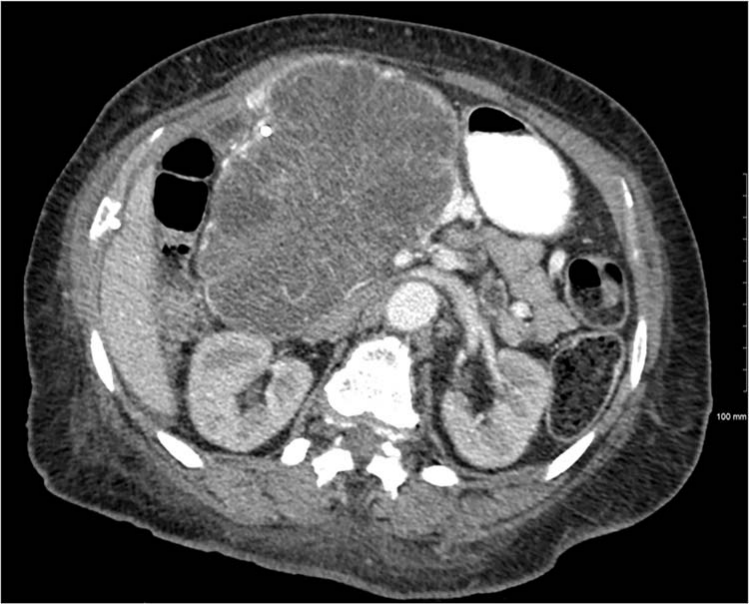
Mass effect to abdominal viscera from the 14 × 10 × 13 cm pancreatic head mass.

Another abdominal CT was performed redemonstrating the large heterogenous mixed attenuating mass within the right abdomen measuring 14.0 × 10.0 × 13.0 cm likely originating from the pancreatic head with resultant displacement of the PTC anteriorly and to the right with extensive pancreatic ductal dilation (Figs 2 and 3).

**Figure 4:**
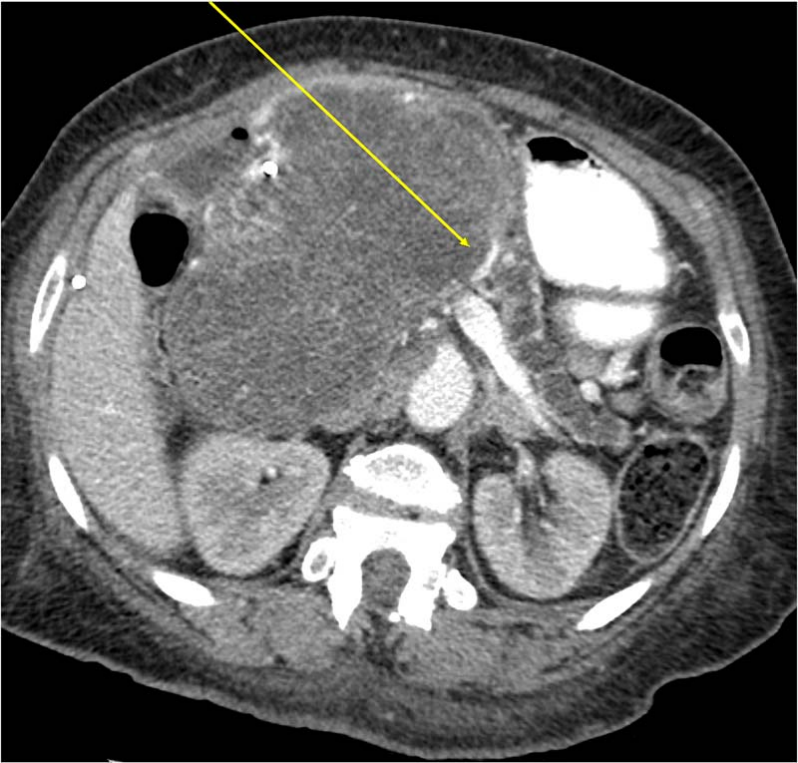
Effacement of SMV (arrow).

The mass extended up into the base of the liver, effaced the portal and superior mesenteric veins (SMV) (Fig. 4), and displaced the hepatic and superior mesenteric arteries.

During this hospitalization, patient was given intravenous resuscitation fluids, started on dialysis, underwent PermCath placement, and exhibited improvement in functional status safe for discharge home with a discharge creatinine of 3.31 mg/dL.

**Figure 5:**
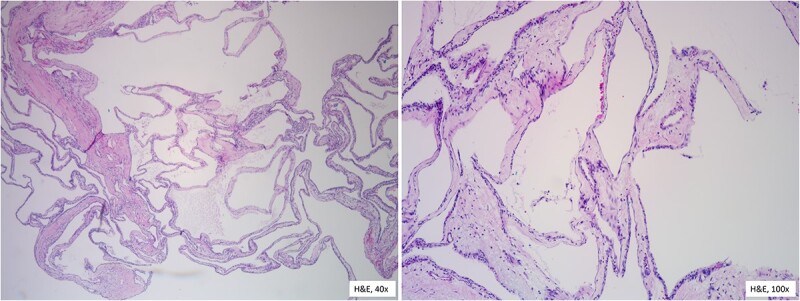
Histopathological features reveal the cysts lined by a single layer of cuboidal cells with clear, glycogen-rich cytoplasm, without atypia.

Two weeks later, the patient presented for pylorus-preserving pancreaticoduodenectomy and peri-portal lymph node dissection. Intraoperatively, the entire pancreas and surrounding structures were distorted. The tumor was closely adhered to the SMV and portal vein. Despite tedious dissection, the SMV had to be repaired with 4–0 Prolene sutures. There was no evidence of other organ or lymph node involvement. The estimated blood loss was 800 cc and patient received blood products during the case. The patient’s postoperative course was uneventful. She was followed in the intensive care unit until postoperative Day 3 and discharged on postoperative Day 8 without any complications. The final pathology report confirmed the diagnosis of benign serous microcystic adenoma measuring 15 cm in diameter with no tumor invasion into the interstitium or vessels. Analysis of the specimen revealed acute bile duct inflammation with mucosal ulceration, chronic pancreatitis, acute and chronic cholecystitis, and subacute and chronic ampullary wall inflammation. Microscopic examination (Fig. 5) of the specimen shows glycogen-rich cysts lined by a single layer of cuboidal or flattened cells. Sectioning through the mass revealed tan-yellow to pink, multicystic cut surface containing thin, brown fluid.

The mass obstructed the main pancreatic duct, did not invade into common bile duct, and adhered to the duodenum, but did not invade it. The patient has followed up in the clinic and has been recovering well from surgery without any acute concerns.

## Discussion

Advances in imaging technologies have led to increased incidental identification of asymptomatic tumors of the pancreas, which makes management challenging. Serous cystadenomas (SCA) of the pancreas are most commonly benign tumors differentiated from mucinous cystadenomas and IPMNs, which should be resected due to their premalignant nature, first described by Compagno and Oertel in 1978: with characteristic innumerable small cysts (< 2 cm) lined by a single row of cuboidal cells with clear cytoplasm [5]. The mean tumor size was 10.8 cm and 71% of patients were symptomatic [5]. Our patient’s tumor was 15 cm in size, and she presented with signs of biliary obstruction. Malignant serous cystadenocarcinomas can be defined by perineural or lymphovascular invasion or metastatic deposits, but can appear histologically indistinguishable from benign tumors [6]. Serous cystadenomas occur most frequently in women during the seventh to eighth decades (our patient was 63 years old in her seventh decade) and are mainly asymptomatic. Nonspecific symptoms have been reported including abdominal pain, nausea, vomiting, and weight loss [3]. Obstructive symptoms like jaundice are more rare, but can be seen with large tumors, similar to how our patient presented in this study. Moreover, lesions located in the pancreatic head and those that are larger in size may have a propensity for more aggressive behavior with adjacent organ or vascular involvement [7]. Our patient’s tumor was adhered to the duodenum and closely associated with the SMV and portal vein. There are five classes of serous cystic neoplasms according to the WHO classification: microcystic, macrocystic (oligocystic), mixed macrocystic and microcystic, von Hippel–Lindau-associated serous cyst, and solid serous adenoma [8]. Microcystic serous cystic neoplasms are the most common and macroscopically are composed of many small cysts with a ‘honeycomb’ appearance. Pathognomonic calcifications with a fibrous central scar are less commonly detected in ~30% of cases [9]. Fibrosis can mimic mural nodules seen in IPMNs, which can complicate diagnosis [10]. Macrocystic serous cystic neoplasms have fewer cysts with thin walls that are larger in size (> 2 cm) [9]. The present case, microscopically, was composed of fibrous pancreatic tissue with numerous small cysts consistent with a pathological diagnosis of microcystic serous adenoma based on the WHO classification system.

Management of these lesions depends on having a secure diagnosis. Correct radiographic diagnosis can be a challenge with serous cystadenomas. Atypical findings of serous cystadenomas include rapid growth, communication with the main pancreatic duct, and large tumors resulting in ductal dilation [8]. Our patient had evidence of the latter two, which have been suggested to exhibit a higher risk for malignant potential [8]. Therefore, these characteristics may potentially confuse a serous cystadenoma for an IPMN, which has different treatment options. Serous cystadenomas seldom have a connection with the main pancreatic duct; therefore, our patient’s tumor could have represented an IPMN or a pseudocyst [10]. Likely, our patient’s lesion compressed the main pancreatic duct extrinsically, leading to main pancreatic duct dilation. Pathology of our specimen described findings of chronic pancreatitis and subacute/chronic ampullary wall inflammation with obstruction of the pancreatic duct. Moreover, imaging techniques consist of CT, MRI, and EUS. CT generally shows a clear, solitary mass, which represents the numerous microcysts with a lobulated appearance, as shown in the present case [6]. CT alone has been reported to be 23% accurate in diagnosing serous cystadenomas [6]. These findings are differentiated from the macrocystic (oligocystic) type featuring lobulated unilocular lesions lacking a central scar and mucinous tumors with peripheral calcifications. EUS-FNA can aid in diagnosis when CT and/or MRI are insufficient in differentiating cystic lesions. However, these aspirates do not necessarily provide a high-level diagnostic accuracy when used alone [8]. In cases of diagnostic uncertainty, like ours, despite having a diagnosis of a serous cystadenoma, there were features that could represent malignant transformation.

**Figure 6:**
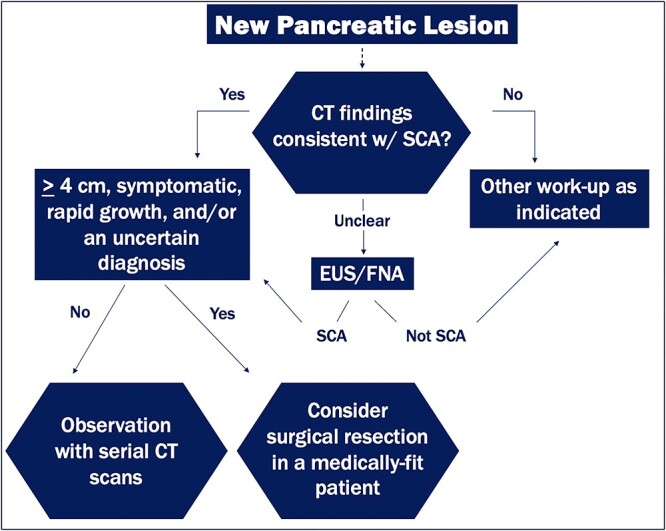
Diagnostic and management algorithm for pancreatic serous cystadenomas.

Overall treatment for pancreatic serous cystadenomas remains controversial despite their benign nature. Some recommend resection for all cystic pancreatic neoplasms, whereas others elect for a more selective approach to treatment. In cases of diagnostic uncertainty, it is important to identify lesions that need to be resected versus ones that can be safely monitored. Surgical resection is curative and reserved for symptomatic serous cystadenomas, tumors ≥ 4 cm, tumors with a rapid growth rate or adjacent organ involvement, and cases with an uncertain diagnosis (Fig. 6).

Early surgical resection, even in the absence of symptoms, is a relatively newer thought based on Tseng *et al.’s* findings that larger tumors are more likely to be symptomatic and have a greater growth rate (2 cm/year) [4]. Palliative surgery may also have a role in terms of quality of life in selected patients (elderly, or high-risk individuals) with large tumors. Distal pancreatectomy is the treatment of choice for pancreatic tumors in the body or tail. Pancreaticoduodenectomy (a Whipple) is the treatment for pancreatic head, or uncinate process tumors. In our case, the patient underwent a Whipple procedure for her symptomatic tumor (causing biliary obstruction) located in the pancreatic head, which initially was concerning for transformation of the serous cystadenoma to a cystadenocarcinoma. The majority agree conservative management is reserved for small (< 4 cm), or asymptomatic serous cystadenomas with close follow-up every 6 months for a 2-year duration and then annually after that (Fig. 6). However, conservative management runs the risk of increased tumor growth and complications, such as biliary obstruction, invasion of surrounding structures, or hemorrhage. Malignant transformation remains a rare event even over long periods of observation [11]. Moreover, some elect for resection with medium size, asymptomatic serous cystadenomas.

## Conclusion

In conclusion, with recent advances in imaging techniques, serous cystadenomas are diagnosed more frequently, and many patients may not have a definitive diagnosis prior to operation, especially with atypical preoperative imaging findings. It appears surgical resection of serous cystadenomas in select patients is warranted. Specifically, in patients with large tumors (≥ 4 cm), rapid growth rate, or an uncertain diagnosis in a medically fit patient. A high degree of diagnostic reliability is crucial in differentiating from other cystic tumors and non-neoplastic cysts due to differences in management. This case highlights a giant serous cystadenoma causing biliary obstruction.

## Data Availability

All data is made available within the case report.
